# Psychiatry and artificial intelligence: A need for informed engagement

**DOI:** 10.4102/sajpsychiatry.v32i0.2668

**Published:** 2026-06-18

**Authors:** Christopher P. Szabo, Carla Kotzé, Lisa Galvin, Yumna Minty, Lihle Mgweba-Bewana, Mvuyiso Talatala, Sisikelelwe Gwanya-Mdletye, Gareth Nortje

**Affiliations:** 1 Department of Psychiatry, Faculty of Health Sciences, University of the Witwatersrand, Johannesburg, South Africa; 2 Department of Psychiatry, Faculty of Health Sciences, University of Pretoria, Pretoria, South Africa; 3 Private, Gqeberha, South Africa; 4 Private, Cape Town, South Africa

The emergence of artificial intelligence (AI) is transforming the healthcare landscape, both clinically and administratively, with the promise of improving patient outcomes. However, as with all innovative technology, the reality of impact emerges over time.^1,2^

The fields of radiology, pathology, gastroenterology and ophthalmology, as well as cardiology, have seen the positive impact of AI systems on screening, diagnosis, prediction and treatment.^3,4^ Aside from these medical specialities benefitting from AI, what of psychiatry? Psychiatry is a holistic discipline, incorporating a bio-psycho-social (and increasingly, spiritual) ethos, which includes a multidisciplinary team approach with a focus on individualised treatment that critically requires an in-depth knowledge of a patient’s life and history. Human interaction is the cornerstone of psychiatric practice, with the therapeutic alliance well established as fundamental to the success of psychotherapy.^5^ However, the South African mental health sector is under-resourced, with a reported 1.52 psychiatrists per 100 000 population, with a distinct urban and private sector, as well as regional bias.^6^ The availability of therapeutic chatbots has been promoted to address increasing need in the face of resource constraints,^7,8^ notwithstanding well-documented instances of suicide associated with AI chatbot use, with appropriate concerns about AI’s ability to handle psychiatric crises appropriately.^9,10^ Most recently, the use of therapeutic chatbots, and of AI, for any psychotherapeutic intervention was banned in Illinois (US).^11^ Furthermore, no AI chatbot has received Food and Drug Administration (FDA) approval as a medical device for therapy.^10^ As stated by Hua et al.,^8^ it is imperative ‘… to ensure that these technologies reliably enhance mental health care’. Accordingly, clinicians need to be fully cognisant of the workings and implications of incorporating AI into their practice.

The lifeblood of AI is data, with massive downward sharing of information by AI into ‘big data’ making it impossible to ascertain the extent to which patients’ information is shared by AI.^12^ While this data sharing is essential for AI to learn, improve and reduce bias, it complicates the process of obtaining informed consent from patients, necessitating that we consider updated ways of obtaining consent, which could include the option of letting patients provide consent each time their information is shared instead of giving a blanket consent.^3,13^ The management of patient data in terms of privacy, security and use needs to be clearly understood. It is essential for patients to understand how AI will be applied to process their information, for both administrative and clinical purposes, as well as how it may affect their healthcare. Informed consent requires a thorough explanation of such use as well as patient understanding, thus respecting patient autonomy.^1,2^

Within the context of data lies a further, crucial issue, that is, the potential for bias and inaccuracy. Specifically, that AI will understand and interpret inputs of data based on whatever data it has been trained on.^1,2^ Under-representation of certain population groups in such data will have the potential for erroneous outputs, potentially impacting not only individual but also population health. In South Africa, the potential to do harm using AI models without adequate diversity of data is a concern, with the risk for bias needing to be carefully considered, and outputs interpreted accordingly with appropriate verification for accuracy. This speaks to the principle of justice. Concerns have been expressed that such bias will perpetuate historical inequality.^14^ Within the context of inequality, the issue of access to AI is germane insofar as the lack of access within disadvantaged communities may further perpetuate inequality. A further, related aspect concerns the language of AI and the need for greater representation of African languages. There is currently such a project underway, and as has been stated, ‘Building AI in our languages is therefore the only way for AI to work for people’.^15^ In addition, the issue of AI governance in Africa has led to a review of both policy and regulatory frameworks with a stated emphasis on the importance of local context to ‘… ensure that the application of AI fosters diverse cultures and languages and supports locally generated data for the benefit of countries and communities …’.^16^

As patients increasingly engage with AI-driven applications, it is essential that clinicians understand these tools’ capabilities and limitations.^10^ This enables clinicians not only to contextualise AI-informed patient narratives but also to contribute to shaping the digital health ecosystem in alignment with ethical and clinical standards. In this regard, the Health Professions Council of South Africa (HPCSA) has published ‘Ethical guidelines on the use of Artificial Intelligence’,^17^ and the Medical Protection Society (MPS) has published the ‘AI safer practice framework’,^18^ with both providing comprehensive guidance for clinicians.

As noted by Sydney Bloch in 2005 ‘… psychiatry is arguably the most person-oriented of all the medical specialities …’.^19^ In the age of AI, such words have never been more prescient. Trust, and ultimately the doctor–patient relationship, is fundamental to clinical outcomes. While technological progress that contributes to improved care should be welcomed, it cannot be at the cost of what is fundamental to the practice of medicine, and psychiatry in particular – trust and the doctor–patient relationship.
